# The mitochondrial genome of the land snail *Cernuella virgata* (Da Costa, 1778): the first complete sequence in the family Hygromiidae (Pulmonata, Stylommatophora)

**DOI:** 10.3897/zookeys.589.7637

**Published:** 2016-05-16

**Authors:** Jun-Hong Lin, Wei-Chuan Zhou, Hong-Li Ding, Pei Wang, Hong-Mu Ai

**Affiliations:** 1 College of Plant Protection, Fujian Agriculture and Forestry University, Fuzhou, Fujian 350002, China; 2 Key Laboratory of Molluscan Quarantine and Identification of AQSIQ, Fujian Entry-Exit Inspection & Quarantine Bureau, Fuzhou, Fujian 350001, China

**Keywords:** DNA sequencing, phylogeny, plant quarantine, secondary structure, white snail

## Abstract

The land snail *Cernuella virgata* (da Costa, 1778) is widely considered as a pest to be quarantined in most countries. In this study, the complete mitochondrial genome of *Cernuella virgata* is published. The mitochondrial genome has a length of 14,147 bp a DNA base composition of 29.07% A, 36.88% T, 15.59% C and 18.46% G, encoding 13 protein-coding genes (PCGs), 22 transfer RNA (tRNA) genes and two ribosomal RNA (rRNA) genes. The complete nucleotide composition was biased toward adenine and thymine, A+T accounting for 69.80%. Nine PCGs and 14 tRNA genes are encoded on the J strand, and the other four PCGs and eight tRNA genes are encoded on the N strand. The genome also includes 16 intergenic spacers. All PCGs start strictly with ATN, and have conventional stop codons (TAA and TAG). All tRNAs fold into the classic cloverleaf structure, except *tRNA^Arg^*, *tRNA^Ser(UCN)^*, *tRNA^Ser(AGN)^* and *tRNA^Pro^*. The first three lack the dihydrouridine arm while the last lacks the TψC arm. There are 502 bp long noncoding regions and 418bp long gene overlaps in the whole mitochondrial genome, accounting for 3.54% and 2.95% of the total length respectively. Phylogenetic analyses based on the sequences of the protein coding genes revealed a sister group relationship between the Hygromiidae and the Helicidae.

protein-coding genes

transfer RNA

ribosomal RNA

## Introduction

The land snail *Cernuella virgata* (da Costa, 1778), also known as the Mediterranean white snail or Common white snail, is endemic to the Mediterranean and western Europe, and has been introduced to America, Australia and Morocco (Barker 2004). The snail is omnivorous, feeding on detritus and plant matter, such as bark, stems and leaves of various green plants. Not only does it destroy agricultural crops, such as beans, cereal, various fruits and vegetables, it also can spread zoonotic food-borne parasitic diseases. For example, the species acts as intermediate host for the terrestrial trematode parasite *Brachylaima cribbi* (Kerney and Cameron 1979; Butcher and Grove 2006). Because of its remarkable adaptability and the severe damage it causes to agriculture, the natural environment and humans, the snail is considered a serious pest in the USA, Australia, Japan, Chile and other countries (Dennis 1996; Barker 2004; USDA 2008; MOA and AQSIQ 2012). One ship carrying barley from Australia was refused entry and berthing by Chile because of the presence of this snail causing huge economic losses (USDA 2008). It is also one of the more important quarantine terrestrial mollusks in America. To prevent invasion and proliferation, the U.S. government has invested considerable human and financial resources to eradicate the snails in Washington, Michigan and North Carolina (USDA 2008). Recently, Chinese ports have intercepted snails in barley, rapeseed and other consignments from abroad. Owing to its great harm, the snail was listed in “The People’s Republic of China entry plant quarantine pest list” by the government in 2012 to prevent its introduction (MOA and AQSIQ 2012).

The metazoan mitochondrial (mt) genome usually comprise 37 genes and some noncoding regions, such as 13 protein coding genes (PCGs) (*COI*−*COIII*, *Cytb*, *ND1*−*ND6*, *ND4L*, *ATP6* and *ATP8*), two ribosomal RNA (rRNA) genes, 22 transfer RNA (tRNA) genes and the AT-rich region or control region (Wolstenholme 1992; Boore 1999). It has been extensively used to study the origin of species, phylogeography and population genetic structure and so on due to its small genome size, fast evolution, uniparental inheritance and lack of extensive recombination (Saccone et al. 1999; Elmerot et al. 2002). To date, only nine species from the order Stylommatophora have been determined as dispersing in Helicidae (Terrett et al. 1995; Groenenberg et al. 2012; Gaitán-Espitia et al. 2013), Bradybaenidae (Yamazaki et al. 1997; Deng et al. 2014), Clausiliidae (Hatzoglou et al. 1995), Succineidae (White et al. 2011), Achatinidae (He et al. 2014) and Camaenidae (Wang et al. 2014). However, there are no reports on the mt genome of the family Hygromiidae. In this work, the complete mt genome of the snail *Cernuella virgata* was obtained firstly using primer walking and shotgun sequencing techniques based on PCR. Studying the mitochondrial genome of *Cernuella virgata* can not only offer more worthwhile information for phylogeny but also be applied to molecular alignment and identification in international plant quarantine measures.

## Materials and methods

### Specimen collection and DNA isolation

Adult snail was intercepted from barley shipments imported to China from southern Australia on 1 March 2012 and stored at -20 °C in the Key Laboratory of Molluscan Quarantine and Identification of AQSIQ, Fujian Entry-Exit Inspection & Quarantine Bureau, Fuzhou, Fujian, China (FJIQBC). Voucher specimens (FJIQBC000123) were deposited in FJIQBC. Total genomic DNA was obtained from approximately 50 mg fresh foot tissue, using the DNeasy Blood and Tissue kit (Qiagen) according to the manufacturer’s instructions.

### DNA sequencing

The entire genome was successfully amplified by polymerase chain reaction (PCR) in overlapping fragments with four pairs of mitochondrial universal primers chosen from previous works (Palumbi et al. 1991; Folmer et al. 1994; Merritt et al. 1998; Hugall et al. 2002) and four pairs of perfectly matched primers designed from sequenced short fragments with Primer Premier 5.0 (Table 1). Short PCRs (< 2 kb) were performed using Takara Taq DNA polymerase (TaKaRa, Dalian, China), with the following cycling conditions: 30s at 94 °C, followed by 35 cycles of 10s at 94 °C, 50s at 40 °C or 45 °C, and 1 min at 72 °C. The final elongation step was continued for 10 min at 72 °C. Long range PCRs (> 4 kb) were performed using Takara Long Taq DNA polymerase (TaKaRa, Dalian, China) under the following cycling conditions: 1 min at 94 °C, followed by 40 cycles of 10s at 98 °C, 50s at 60 °C, 4−8 min at 68 °C, and the final elongation step at 72 °C for 6 min. The PCR products were checked by spectrophotometry and 1.0% agarose gel electrophoresis.

**Table 1.** Primer pairs used for PCR amplification.

No. of fragment	Primer name	Nucleotide sequence (5’ – 3’) and location	Size (bp)	Reference
1	LCO-1490	GGTCAACAAATCATAAAGATATTGG		Folmer et al. 1994
	HCO-2198	TAAACTTCAGGGTGACCAAAAAATCA		Folmer et al. 1994
2	F1231	GAACGGGTTAGTTTGTTTGTCT(490–511)	1763	Present study
	R1231	TAGGGTCTTCTCGTCTATTATGGT(2229–2252)		Present study
3	16Sar-L	CGCCTGTTTATCAAAAACAT		Palumbi et al. 1991
	16Sbr-H	CCGGTCTGAACTCAGATCACGT		Palumbi et al. 1991
4	123F116	TGTAACCATAATAGACGAGAAGACC(2225–2249)	4545	Present study
	123R1b	TAGGAGCAAAAAATACTACCAGAAA(6745–6769)		Present study
5	144F	TGAGSNCARATGTCNTWYTG		Merritt et al. 1998
	272R	GCRAANAGRAARTACCAYTC		Merritt et al. 1998
6	123Fb	CTTTTCACCCCTACTTTAC(6683–6701)	1044	Present study
	123RII	ACTCCCTTTCAGGTGTTAT(7708–7726)		Present study
7	FCOII	AAATAATGCTATTTCATGAYCAYG		Hugall et al. 2002
	RCOII	GCTCCGCAAATCTCTGARCAYTG		Hugall et al. 2002
8	F1233	AGTTACATTGGCCCTCCCTAGTCTTCGC(7560–7587)	6930	Present study
	R1233	GTAAACGGTTCAACCTGTACCAGCTCCC(315–342)		Present study

The BigDye Terminator Sequencing Kit (Applied Biosystems, San Francisco, CA, USA) and the ABI PRIMER^TM^ 3730XL DNA Analyzer (PE Applied Biosystems) were used to sequence short fragments from both directions after purification. For the long fragments, the shotgun libraries of *Cernuella virgata* were constructed, and the positive clones were then sequenced using the above kit and sequenator with vector-specific primers *Bca*Best primer M13-47 and *Bca*Best Primer RV-M.

### Genome annotation and inference of secondary structure

To control sequencing errors, each partial sequence was evaluated at least twice. Annotations and editing procedures of the mitochondrial genomes of *Cernuella virgata* were performed in MEGA5.0. Mitochondrial PCGs and rRNA genes were identified by BLAST searches at NCBI against other Eupulmonata sequences (Wang et al. 2014; He et al. 2014; Deng et al. 2014; Yang et al. 2014). The limits of both protein coding and rRNA genes were adjusted manually based on location of adjacent genes, and the presence of start and stop codons. The tRNA genes were located using DOGMA (Wyman et al. 2004) and tRNAscan-SE v.1.21(Lowe and Eddy 1997), while others that could not be determined by DOGMA and tRNAscan-SE were identified by comparison with other land snails (Terrett et al. 1995; Yamazaki et al. 1997; Groenenberg et al. 2012; Gaitán-Espitia et al. 2013; Wang et al. 2014).

The base composition and codon usage were analyzed with MEGA 5.0 (Tamura et al. 2007). AT skew and GC skew were used to describe strand asymmetry according to the formulae AT = [A−T]/[A+T] and GC = [G−C]/[G+C] (Perna and Kocher 1995).

### Phylogenetic analyses

Phylogenetic analyses were performed based on 15 complete mt genomes of gastropods from GenBank (Table 2) using maximum likelihood (ML) method. Two species from Basommatophora and Opisthobranchia were selected as outgroups. A DNA alignment with 10,362 bp length was inferred from the amino acid alignment of 13 PCGs using MEGA 5.0 (Tamura et al. 2007). The selection of best-fit-substitution model for ML estimation was performed using MEGA 5.0 with corrected Akaike information criterion (AIC). Node supports for ML analyses were calculated through 1000 bootstrap replicates. All other settings were kept as default.

**Table 2.** Summary of samples used in this study.

Subclass /order	Family	Species	Accession number	Reference
Stylommatophora	Hygromiidae	*Cernuella virgata*	KR736333	Present study
	Camaenidae	*Camaena cicatricosa*	KM365408	Wang et al. 2014
		*Camaena* sp.	KT001074	Ding et al. 2015 (submitted)
	Bradybaenidae	*Euhadra herklotsi*	Z71693 – Z71701	Yamazaki et al. 1997
		*Mastigeulota kiangsinensis*	KM083123	Deng et al. 2014
		*Aegista diversifamilia*	KR002567.1	Huang et al. 2015
		*Dolicheulota formosensis*	KR338956.1	Huang et al. 2015
	Helicidae	*Cornu aspersum*	JQ417195	Gaitán-Espitia et al. 2013
		*Cepaea nemoralis*	CMU23045	Terrett et al. 1995
		*Cylindrus obtusus*	JN107636	Groenenberg et al. 2012
	Succineidae	*Succinea putris*	JN627206	White et al. 2011
	Clausiliidae	*Albinaria caerulea*	X83390	White et al. 2011
	Achatinidae	*Achatina fulica*	NC024601	He et al. 2014
Basommatophora	Lymnaeidae	*Galba pervia*	JN564796	Liu et al. 2012
Opisthobranchia	Aplysiidae	*Aplysia californica*	AY569552	Knudsen et al. 2006

## Results

### Genome structural features

**Figure 1.**
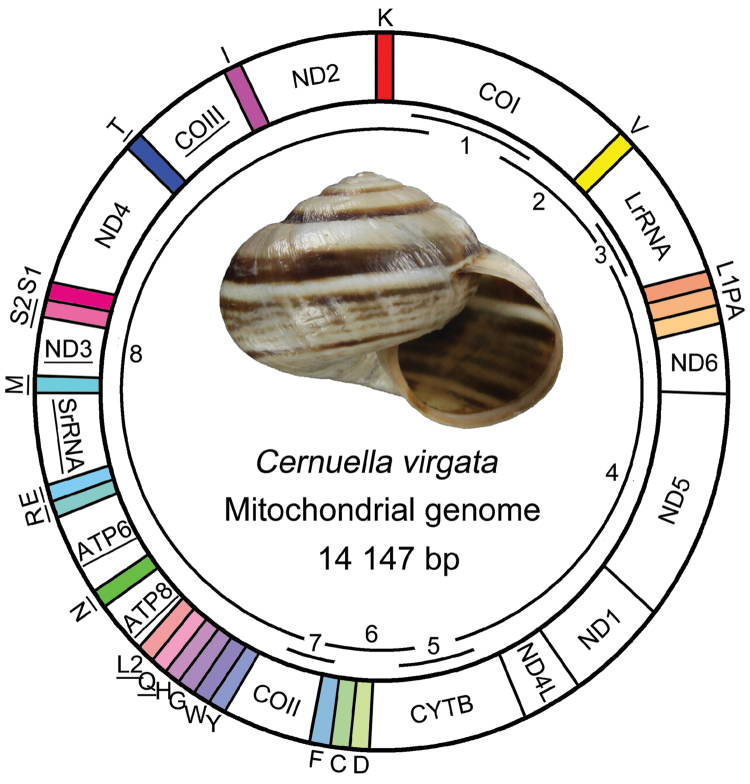
The mt genome of *Cernuella virgata*. The tRNA genes are labeled based on the IUPACIUB single letter amino acid codes. Genes with underline illustrate the direction of transcription from 3’ to 5’, and without underline revealing from 5’ to 3’. Numbers and overlapping lines within the circle indicate PCR fragments amplified for sequencing (see Table 1).

The entire circular genome was 14,147 bp in length (GenBank: KR736333), containing 13 PCGs, 22 tRNA genes and two rRNA genes (Figure 1). Twenty-four genes were encoded on the majority coding strand (J strand) while 13 genes were encoded on the minority coding strand (N strand) (*tRNA^Gln^*, *tRNA^Leu(UUR)^*, *tRNA^Asn^*, *tRNA^Arg^*, *tRNA^Glu^*, *tRNA^Met^*, *tRNA^Ser(UCN)^*, *tRNA^Thr^*, *ATP6*, *ATP8*, *ND3*, *COIII* and *SrRNA*) (Table 3). The nucleotide composition of the whole genome was biased toward adenine and thymine, accounting for 69.80% of base composition (Table 4). Gene overlaps with a total of 418 bp have been found at 14 gene junctions; the longest overlap (85 bp) existed between *ND5* and *ND1*. In addition, 502 nucleotides were dispersed in 16 intergenic spacers, the largest of which was 149 bp long between *tRNA^Trp^* and *tRNA^Gly^*. Additionally, two long spacers of 77 bp and 76 bp each were found between *ND4L* and *ND1*, *tRNA^Ser(UCN)^* and *tRNA^Ser(AGN)^*, respectively. There were seven close gene junctions with no intergenic spacers or overlap (Table 3).

**Table 3.** Organization of the *Cernuella virgata* mt genome.

Gene	Direction	Location	Size (bp)	Anticodon	Start codon	Stop codon	Intergenic nucleotides
*COI*	F	1–1497	1497		ATT	TAA	26
*tRNA^Val^*	F	1494–1554	61	1524–1526 TAC			–4
*lrRNA*	F	1555–2567	1013				0
*tRNA^Leu(CUN)^*	F	2568–2628	61	2597–2599 TAG			0
*tRNA^Pro^*	F	2629–2685	57	2655–2657 TGG			0
*tRNA^Ala^*	F	2687–2748	62	2718–2720 TGC			1
*ND6*	F	2767–3222	456		ATA	TAA	18
*ND5*	F	3227–4888	1662		ATT	TAA	4
*ND1*	F	4804–5769	966		ATG	TAG	–85
*ND4L*	F	5847–6215	369		ATT	TAA	77
*CytB*	F	6151–7167	1017		ATA	TAG	–65
*tRNA^Asp^*	F	7157–7214	58	7188–7190 GTC			–11
*tRNA^Cys^*	F	7215–7276	62	7245–7247 GCA			0
*tRNA^Phe^*	F	7283–7341	59	7313–7315 GAA			6
*COII*	F	7387–8031	645		ATT	TAA	45
*tRNA^Tyr^*	F	8015–8083	69	8046–8048 GTA			–17
*tRNA^Trp^*	F	8071–8132	62	8102–8104 TCA			–13
*tRNA^Gly^*	F	8282–8341	60	8311–8313 TCC			149
*tRNA^His^*	F	8338–8398	61	8369–8371 GTG			–4
*tRNA^Gln^*	R	8400–8457	58	8427–8429 TTG			1
*tRNA^Leu(UUR)^*	R	8457–8513	57	8485–8487 TAA			–1
*ATP8*	R	8485–8754	270		ATG	TAA	–29
*tRNA^Asn^*	R	8743–8804	62	8771–8773 GTT			–12
*ATP6*	R	8807–9472	666		ATG	TAA	2
*tRNA^Arg^*	R	9458–9517	60	9489–9491 TCG			–15
*tRNA^Glu^*	R	9518–9578	61	9547–9549 TTC			0
*SrRNA*	R	9579–10277	699				0
*tRNA^Met^*	R	10278–10343	66	10306–10308 CAT			0
*ND3*	R	10304–10735	432		ATA	TAA	–40
*tRNA^Ser(UCN)^*	R	10691–10743	53	10723–10725 TGA			–45
*tRNA^Ser(AGN)^*	F	10820–10880	61	10844–10846 GCT			76
*ND4*	F	10904–12178	1275		ATT	TAG	23
*tRNA^Thr^*	R	12182–12246	65	12210–12212 TGT			3
*COIII*	R	12170–13051	882		ATG	TAA	–77
*tRNA^Ile^*	F	13068–13127	60	13096–13098 GAT			16
*ND2*	F	13182–14060	879		ATA	TAG	54
*tRNA^Lys^*	F	14062–14121	60	14090–14092 TTT			1

Note: Negative numbers indicate adjacent gene overlap.

### Protein coding genes

The total length of all PCGs was 10, 977 bp, accounting for 77.59% of the entire mt genome (Table 4). All PCGs started strictly with the Start Codon ATN (four with ATG, five with ATT, and four with ATA) and ended with the conventional stop codons TAA or TAG. (Table 3).

**Table 4.** Nucleotide composition and skewness of the *Cernuella virgata* mt genome.

	Proportion of nucleotides							
Feature	%A	%T	%G	%C	%A+T	AT Skew	GC Skew	No. of nucleotides
Whole genome	29.07	36.88	18.46	15.59	69.80	–0.12	0.08	14147
Protein coding genes	26.39	39.31	18.43	15.87	69.26	–0.20	0.07	10977
Protein coding genes (J)	26.08	39.96	18.70	15.26	69.17	–0.21	0.10	8739
Protein coding genes (N)	27.61	36.77	17.38	18.23	69.67	–0.14	–0.02	2034
tRNA genes	31.46	34.23	18.73	15.58	71.41	–0.04	0.09	1335
tRNA genes (J)	29.82	34.77	20.30	15.10	70.77	–0.08	0.15	788
tRNA genes (N)	33.82	33.46	16.45	16.27	72.54	0.01	0.01	547
rRNA genes	32.83	35.63	17.00	14.54	72.42	–0.04	0.08	1712

**Figure 2.**
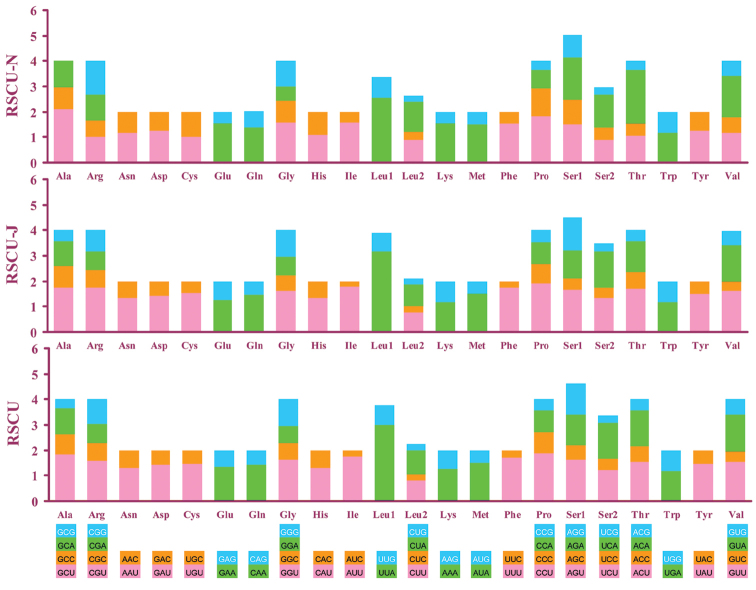
Relative synonymous codon usage (RSCU) in the *Cernuella virgata* mt genome. Codon families are provided on the x axis.

Codon usage could reveal nucleotide bias. NNA and NNU as codons were used frequently in most PCGs. Additionally, the codons TTT (phenylalanine), TTA (leucine) and ATT (isoleucine) composing A and T were used widely (Figure 2).

### Transfer RNA genes

**Figure 3.**
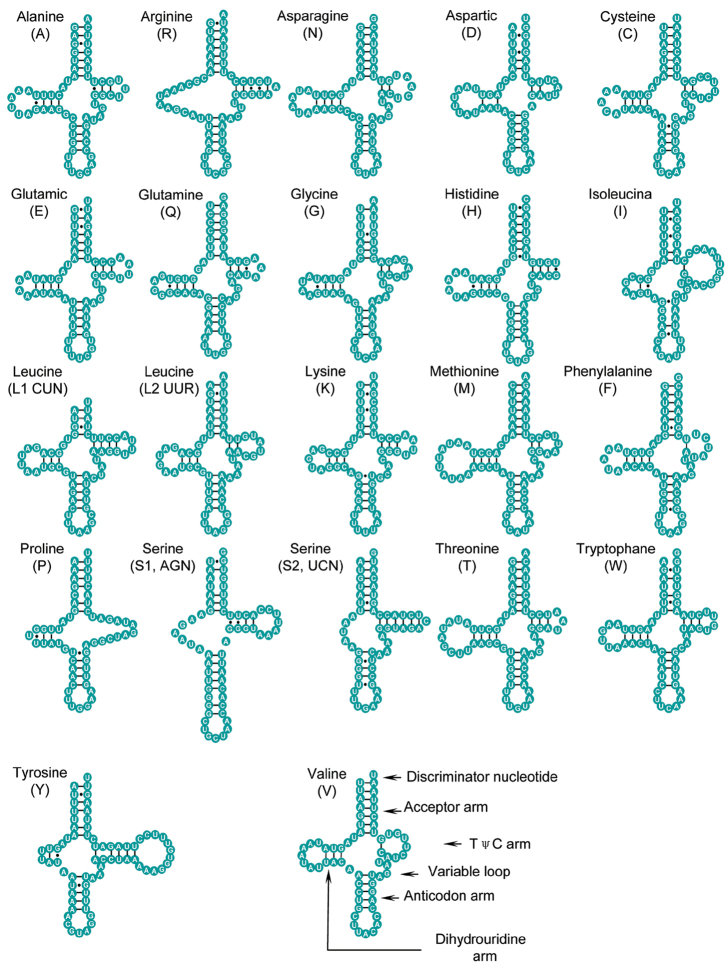
Inferred secondary structures of 22 tRNA genes in *Cernuella virgata*. Dashes (-) indicate Watson-Crick base pairing and bullets (•) indicate G-U base pairing.

The length of tRNA genes ranged from 53 to 69 bp.The 22 tRNA genes typically found in metazoan mt genomes were also discovered in *Cernuella virgata*; eleven of them were determined by tRNAscan-SE and eight of them were determined by DOGMA. Another three tRNA genes that could not be detected by the above two programs were identified and passed through comparisons with known patterns of previous research Fourteen tRNA genes were encoded on the J strand and the remainder on the N strand. Most tRNA genes could be folded into classic clover leaf structures except for *tRNA^Arg^*, *tRNA^Ser(UCN)^* and *tRNA^Ser(AGN)^*, which lack the dihydrouridine arm. The gene *tRNA^Pro^* has a loop in its TψC arm (Figure 3).

In some tRNA genes, non-Watson-Crick matches and aberrant loops had been found. For example, a total of 41 unmatched base pairs existed in some tRNAs, and 18 of them were G-U non-classical pairs, most of which existed in Discriminator nucleotide, anticodon arm and Dihydrouridine arm (Figure 3).

### Ribosomal RNA genes

The rRNA genes of *Cernuella virgata* encompassed the *lrRNA* and *srRNA* genes with a length of 1,013 bp and 699 bp, repsectively. The former was situated between *tRNA^Val^* and *tRNA^Leu(CUN)^* and the latter was located between *tRNA^Glu^* and *tRNA^Met^* (Table 3).

### Noncoding regions

In the mitochondrial genome of *Cernuella virgata*, there are 16 noncoding regions with total 502 bp length, accounting for 3.54%. The longest was 149bp, between *tRNA^Trp^* and *tRNA^Gly^*. The shortest was 1 bp existing three regions, respectively locating *tRNA^Pro^* and *tRNA^Ala^*, *tRNA^His^* and *tRNA^Gln^*, *ND2* and *tRNA^Lys^* (Table 3).

### Phylogenetic reconstruction

**Figure 4.**
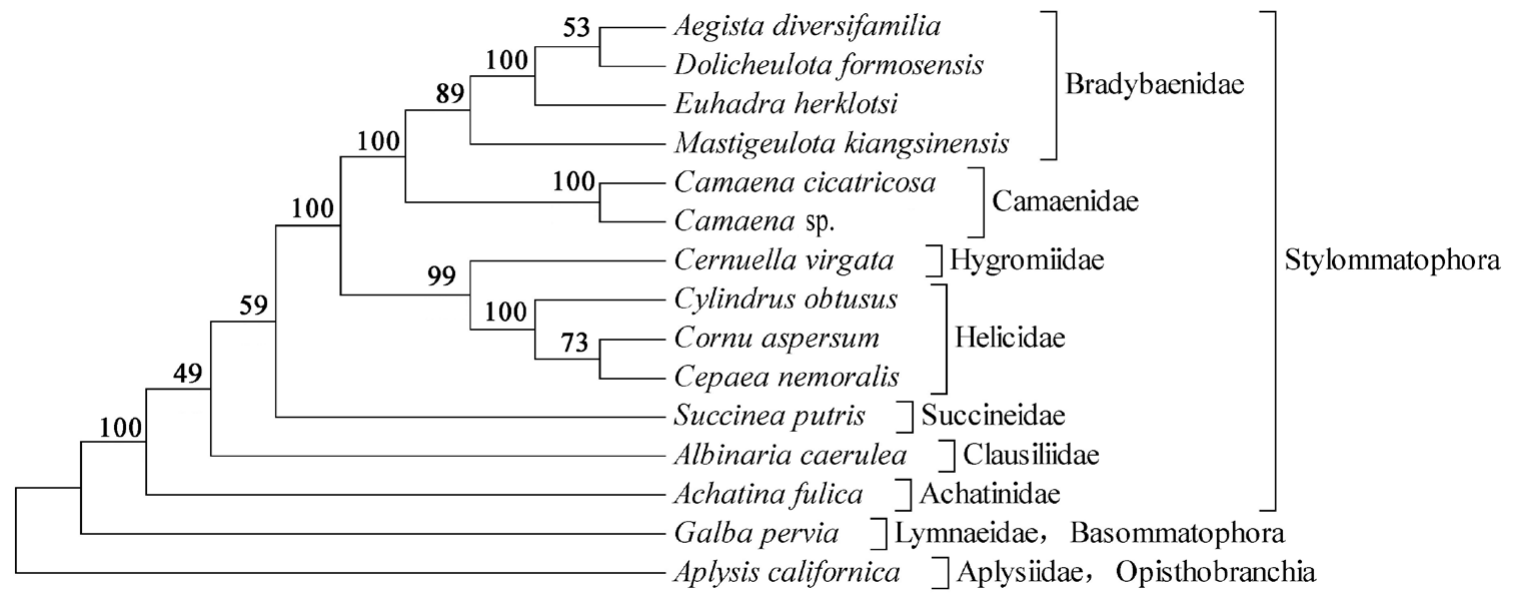
Phylogenetic tree inferred by maximum likelihood (ML) method based on 13 protein genes. The tree is rooted with *Aplysis californica* and *Galba pervia*. Numbers on the nodes represent bootstrap values.

The ML tree (Figure 4) presented nine major clades containing the families Helicidae, Hygromiidae, Camaenidae, Bradybaenidae, Succineidae, Clausiliidae, Achatinidae, Lymnaeidae and Aplysiidae. The four bradybaenid species and three helicid species each formed a clade and a sister pair. In addition, we found that Camaenidae and Bradybaenidae each were monophyletic and also in a sister group relationship with each other.

## Discussion

The length of mt genome of *Cernuella virgata* was 304 bp longer than *Camaena cicatricosa* and 97 bp longer than *Cornu aspersum*. All gene directions showed similarity to the sequenced mt genome of *Camaena cicatricosa*, but gene order was different, especially with respect to the positions between *CYTB* and *ATP8* genes (Gaitán-Espitia et al. 2013; Wang et al. 2014). The overall mt genome of *Cernuella virgata* was loose particularly, with more and longer intergenic spacers.

In the study of mt genome of *Camaena cicatricosa*, GTG is the start codon of the *COII* gene, and *COI* and *ND6* genes of *Cornu aspersum* start with TTG (Gaitán-Espitia et al. 2013; Wang et al. 2014). From previous studies we can see that most start signals of land snails were consistent with *Cernuella virgata* factually, but ATC, TTA, TTG, CTT, TCG and CGA as start signals have been found (Raay and Crease 1994; Crease 1999; Yamazaki et al. 1997; Yu et al. 2007; Groenenberg et al. 2012; Gaitán-Espitia et al. 2013; Wang et al. 2014). Conventional stop codons TAA and TAG have been found in all PCGs of *Cernuella virgata*, which corresponds to *Camaena cicatricosa* (Wang et al. 2014). However, *COII*, *CYTB*, *ND3* and *ATP8* genes of *Cornu aspersum* from the family Helicidae ended with T, and this phenomenon has also been discovered in other snails as well (Terrett et al. 1995; Hatzoglou et al. 1995; Yamazaki et al. 1997; White et al. 2011; Groenenberg et al. 2012; Wang et al. 2012; Gaitán-Espitia et al. 2013). Some authors suggested that this nucleotide exchange was caused by post-transcriptional polyadenylation (Ojala et al. 1981; Cha et al. 2007).

Usually, in the tRNA, the Acceptor arm (7 bp) and Anticodon arm (5 bp) were conservative in size (Kinouchi et al. 2000). However, the length of Acceptor arm of *tRNA^Leu(CUN)^* in *Cernuella virgata* was distinctive, with only 4 bp in size. The Anticodon arm of tRNA^Ser(AGN)^ (8 bp) and all Anticodon loops (7 nucleotides) was coincident with the snail *Camaena cicatricosa* (Wang et al. 2014). The remaining arms and loops changed apparently in size comparing to that of other land snails (Hatzoglou et al. 1995; Groenenberg et al. 2012; Wang et al. 2014). Some non-Watson-Crick matches existed in all tRNA, including G-U pairs, A-C mismatch, U-C mismatch etc. Tomita et al. (2001) raised that these mismatches may can be rectified by post-transcriptional RNA-editing mechanism to hold tRNA function.

Noncoding regions are assumed to splice recognition sites during the process of transcription (He et al. 2005). In the previous sequenced complete mt genome of the order Stylommatophora, the noncoding regions range from 1 bp to 65 bp (Hatzoglou et al. 1995; Terrett et al. 1995; Yamazaki et al. 1997; White et al. 2011; Groenenberg et al. 2012; Gaitán-Espitia et al. 2013; Deng et al. 2014; Wang et al. 2014) except *Achatina fulica* with 551 bp length (He et al. 2014). In metazoan mt genomes, these noncoding regions are normal. The longest one can be called control region or AT-rich region (Boore 1999). Usually, changes in length of the whole mt genome are mainly caused by difference of the control region (Zhang and Hewitt 1997). However, the control region may not be aligned accurately in gastropods (Groenenberg et al. 2012) except in *Achatina fulica* which included a 551 bp putative control region (POR) between *COI* and *tRNA^Val^* (He et al. 2014). Another ten sequenced stylommatophoran species may possess short putative control region located in different places (Hatzoglou et al. 1995; Terrett et al. 1995; Yamazaki et al. 1997; White et al. 2011; Groenenberg et al. 2012; Gaitán-Espitia et al. 2013; Deng et al. 2014; Wang et al. 2014; Huang et al. 2015; 2015). The PORs of *Cernuella virgata*, *Mastigeulota kiangsinensis* and *Dolicheulota formosensis* are situated adjacent to *tRNA^Trp^*, at 149 bp, 216 bp and 245 bp respectively. The PORs of *Camaena cicatricosa* (29 bp) and *Succinea putris* (48 bp) were located between *COIII* and *tRNA^Ile^*. Two other helicid species had PORs located between *COIII* and *tRNA^Ser^* with lengths of 158–186 bp, whereas the PORs of *Albinaria caerulea* (65 bp), *Aegista diversifamilia* (93 bp), *Cylindrus obtusus* (395 bp) and *Euhadra herklotsi* (78 bp) were specific, respectively between *ND3* and *tRNA^Ser^*, *tRNA^Met^ and tRNA^Ser^*, *ND5* and *tRNA^Ala^*, *tRNA^Se r (UCN)^* and *tRNA^Ser(AGN)^*. The absence of a control region was consistent with other gastropods (Deng et al. 2014; Wang et al. 2014; Yang et al. 2014). In the present study, the longest noncoding region was 149 bp, which was the second longest one by far.

Three species in the Helicidae were sister groups and consistent with previous works (Gaitán-Espitia et al. 2013). However, the systematics of Camaenidae, Helicidae and Bradybaenidae are complicated and have not been fully resolved; systematic and phylogenetic studies based on analyses of morphological and molecular markers have produced inconsistent results (Scott 1996; Cuezzo 2003; Wade et al. 2007; Hirano et al. 2014). More complete taxon sampling need to be prepared to assess the phylogenetic relationship of these three families.

## References

[B1] Barker GM (2004) Natural Enemies of Terrestrial Molluscs. CABI Publishing, London, 644 pp. doi: 10.1079/9780851993195.0000

[B2] Boore JL (1999) Animal mitochondrial genomes. Nucleic Acids Research 27(8): 1767–1780. doi: 10.1093/nar/27.8.1767 10101183 10.1093/nar/27.8.1767 PMC148383

[B3] Boore JL (2006) The complete sequence of the mitochondrial genome of *Nautilus macromphalus* (Mollusca: Cephalopoda). BMC Genomics 7: . doi: 10.1186/1471-2164-7-182 10.1186/1471-2164-7-182 PMC1544340 16854241

[B4] Butcher AR Grove DI (2006) Seasonal variation in rates of sporocyst and metacercarial infection by *Brachylaima cribbi* in helicid and hygromiid land snails on the Yorke Peninsula, South Australia. Australian Journal of Zoology 53(6): 375–382. doi: 10.1071/ZO05054

[B5] Cha SY Yoon HJ Lee EM Yoon MH Hwang JS Jin BR Han YS Kim I (2007) The complete nucleotide sequence and gene organization of the mitochondrial genome of the bumblebee, *Bombus ignitus* (Hymenoptera: Apidae). Gene 392: 206–220. doi: 10.1016/j.gene.2006.12.031 17321076 10.1016/j.gene.2006.12.031

[B6] Crease TJ (1999) The complete sequence of the mitochondrial genome of *Dapnia pulex* (Cladocera: Crustacea). Gene 233: 89–99. doi: 10.1016/S0378-1119(99)00151-1 10375625 10.1016/s0378-1119(99)00151-1

[B7] Deng PJ Wang WM Huang XC Wu XP Xie GL Ouyang S (2016) The complete mitochondrial genome of Chinese land snail *Mastigeulota kiangsinensis* (Gastropoda: Pulmonata: Bradybaenidae). Mitochondrial DNA 27(2): 1084–1085. doi: 10.3109/19401736.2014.953083 25185698 10.3109/19401736.2014.953083

[B8] Dennis H (1996) Snails: A Quarantine Concern for Export Citrus to the USA. Entomology Unit, SARDI, Australia.

[B9] Elmerot C Arnason U Gojobori T (2002) The mitochondrial genome of the pufferfish, Fugu rubripes, and ordinal teleostean relationships. Gene 295(2): 163–172. doi: 10.1016/s0378-1119(02)00688-1 12354650 10.1016/s0378-1119(02)00688-1

[B10] Folmer O Black M Hoeh W Lutz R Vrijenhoek R (1994) DNA primers for amplification of mitochondrial cytochrome c oxidase subunit I from diverse metazoan invertebrates. Molecular Marine Biology and Biotechnology 3: 294–299. 7881515

[B11] Gaitán-Espitia JD Scheihing R Poulin E Artacho P Nespolo RF (2013) Mitochondrial phylogeography of the land snail *Cornu aspersum*: tracing population history and the impact of human-mediated invasion in austral South America. Evolutionary Ecology Research 15: 1–18.

[B12] Grande C Templado J Cervera JL Zardoya R (2002) The complete mitochondrial genome of the nudibranch *Roboastra europaea* (Mollusca: Gastropoda) supports the monophyly of opisthobranchs. Molecular Biology and Evolution 19(10): 1672–1685. doi: 10.1093/oxfordjournals.molbev.a003990 12270894 10.1093/oxfordjournals.molbev.a003990

[B13] Groenenberg DSJ Pirovano W Gittenberger E Schilthuizen M (2012) The complete mitogenome of *Cylindrus obtusus* (Helicidae, Ariantinae) using Illumina next generation sequencing. BMC Genomics 13: . doi: 10.1186/1471-2164-13-114 10.1186/1471-2164-13-114 PMC3474148 22448618

[B14] Hatzoglou E Rodakis GC Lecanidou R (1995) Complete sequence and gene organization of the mitochondrial genome of the land snail *Albinaria coerulea*. Genetics 140(4): 1353–1366. 7498775 10.1093/genetics/140.4.1353 PMC1206699

[B15] He Y Jones J Armstrong M Lamberti F Moens M (2005) The mitochondrial genome of *Xiphinema americanum* *sensu stricto* (Nematoda: Enoplea): Considerable economization in the length and structural features of encoded genes. Journal of Molecular Evolution 61: 819–833. doi: 10.1007/s00239-005-0102-7 16315110 10.1007/s00239-005-0102-7

[B16] He ZP Dai XB Zhang S Zhi TT Lun ZR Wu ZD Yang TB (2014) Complete mitochondrial genome of the giant African snail, *Achatina fulica* (Mollusca: Achatinidae): a novel location of putative control regions (CR) in the mitogenome within Pulmonate species. Mitochondrial DNA 27(2): 1–2. 24975387 10.3109/19401736.2014.930833

[B17] Hirano T Kameda Y Kimura K Chiba S (2014) Substantial incongruence among the morphology, taxonomy, and molecular phylogeny of the land snails *Aegista*, *Landouria*, *Trishoplita*, and *Pseudobuliminus* (Pulmonata: Bradybaenidae) occurring in East Asia. Molecular Phylogenetics and Evolution 70: 171–181. doi: 10.1016/j.ympev.2013.09.020 24096054 10.1016/j.ympev.2013.09.020

[B18] Hugall A Moritz C Moussalli A Stanisic J (2002) Reconciling paleodistribution models and comparative phylogeography in the Wet Tropics rainforest land snail *Gnarosophia bellendenkerensis* (Brazier 1875). PNAS 99(9): 6112–6117. doi: 10.1073/pnas.092538699 11972064 10.1073/pnas.092538699 PMC122911

[B19] Kerney MP Cameron RAD (1979) A field guide to the land snails of Britain and northwestern Europe. London, Collins, 288 pp.

[B20] Kinouchi M Kanaya S Ikemura T Kudo Y (2000) Detection of tRNA based on the cloverleaf secondary structure. Genome Information 11: 301–302.

[B21] Lowe TM Eddy SR (1997) TRNAscan-SE: a program for improved detection of transfer RNA genes in genomic sequence. Nucleic Acids Research 25: 955–964. doi: 10.1093/nar/25.5.0955 9023104 10.1093/nar/25.5.955 PMC146525

[B22] Merritt TJS Shi L Chase MC Rex MA Etter RJ Quattro JM (1998) Universal cytochrome b primers facilitate intraspecific studies in molluscan taxa. Molecular Marine Biology and Biotechnology 7: 7–11. 9597773

[B23] MOA, AQSIQ (2012) The List of Quarantine Pests of the People’s Republic of China of the Entry of Plants. Beijing, China.

[B24] Ojala D Montoya J Attardi G (1981) tRNA punctuation model of RNA processing in human mitochondrial. Nature 290(5806): 470–474. doi: 10.1038/290470a0 7219536 10.1038/290470a0

[B25] Palumbi S Martin A Romano S Mcmillan WO Stice L Grabowwski G (1991) The Simple Fool’s Guide to PCR. Department of Zoology, University of Hawaii, Honolulu.

[B26] Perna NT Kocher TD (1995) Patterns of nucleotide composition at fourfold degenerate sites of animal mitochondrial genomes. Journal of Molecular Evolution 41: 353–358. doi: 10.1007/BF01215182 7563121 10.1007/BF00186547

[B27] Raay TJ Crease TJ (1994) Partial mitochondrial DNA sequence of the crustacean *Daphnia pulex*. Current Genetics 25: 66–72. doi: 10.1007/BF00712970 8082168 10.1007/BF00712970

[B28] Saccone C De GC Gissi C Pesole G Reyes A (1999) Evolutionary genomics in Metazoa: the mitochondrial DNA as a model system. Gene 238(1): 195–209. doi: 10.1016/S0378-1119(99)00270-X 10570997 10.1016/s0378-1119(99)00270-x

[B29] Scott B (1996) Phylogenetic relationships of the Camaenidae. Journal of Molluscan Studies 62: 65–73. doi: 10.1093/mollus/62.1.65

[B30] Steinke D Albrecht C Pfenninger M (2004) Molecular phylogeny and character evolution in the Western Palearctic Helicidae s.l. (Gastropoda: Stylommatyophora). Molecular Phylogenetics and Evolution 32: 724–734. doi: 10.1016/j.ympev.2004.03.004 15288050 10.1016/j.ympev.2004.03.004

[B31] Tamura K Peterson D Stecher G Nei M Kumar S (2011) MEGA5: molecular evolutionary genetics analysis using maximum likelihood, evolutionary distance, and maximum parsimony methods. Molecular Biology and Evolution 28: 2731–2739. doi: 10.1093/molbev/msr121 21546353 10.1093/molbev/msr121 PMC3203626

[B32] Terrett JA Miles S Thomas RH (1995) Complete DNA sequence of the mitochondrial genome of *Cepaea nemoralis* (Gastropoda: Pulmonata). Journal of Molecular Evolution 42: 160–168. doi: 10.1007/BF02198842 8919868 10.1007/BF02198842

[B33] USDA (2008) Port of Tacoma *Cernuella virgata* (*C. virgata*) Eradication Program in Pierce County, Washington, 12 pp.

[B34] Wade CM Hudelot C Davison A Naggs F Mordan PB (2007) Molecular phylogeny of the helicoid land snails (Pulmonata: Stylommatophora: Helicoidea), with special emphasis on the Camaenidae. Journal of Molluscan Studies 73: 411–415. doi: 10.1093/mollus/eym030

[B35] Wang JP Nie XP Cao TW Zhang M Guo YP Ma EB Zhang XN (2012) Analysis of complete mitochondrial genome of *Sasakia charonda coreana* (Lepidoptera, Nymphalida). Acta Zootaxonomica Sinica 37(1): 1–9.

[B36] Wang P Yang HF Zhou WC Hwang CC Zhang WH Qian ZX (2014) The mitochondrial genome of the land snail *Camaena cicatricosa* (Müller, 1774) (Stylommatophora: Camaenidae): Presently known as the first in Camaenidae. ZooKeys 451: 33–48. doi: 10.3897/zookeys.451.8537 25493046 10.3897/zookeys.451.8537 PMC4258619

[B37] White TR Conrad MM Tseng R Balayan S Golding R Martins AM Dayrat BA (2011) Ten new complete mitochondrial genomes of pulmonates (Mollusca: Gastropoda) and their impact on phylogenetic relationships. BMC Evolutionary Biology 11(1): . doi: 10.1186/1471-2148-11-295 10.1186/1471-2148-11-295 PMC3198971 21985526

[B38] Wyman SK Jansen RK Boore JL (2004) Automatic annotation of organellar genomes with DOGMA. Bioinformatics 20(17): 3252–3255. doi: 10.1093/bioinformatics/bth352 15180927 10.1093/bioinformatics/bth352

[B39] Yamazaki N Ueshima R Terrett JA Yokobori S Kaifu M Segawa R Kobayashi T Numachi K Ueda T Nishikawa K Watanabe K Thomas RH (1997) Evolution of Pulmonate Gastropod mitochondrial genomes: comparisons of gene organizations of *Euhadra*, *Cepaea* and *Albinaria* and implications of unusual tRNA secondary structures. Genetics 145: 749–758. 9055084 10.1093/genetics/145.3.749 PMC1207859

[B40] Yang HR Zhang JE Luo H Luo MZ Guo J Deng ZX Zhao BL (2014) The complete mitochondrial genome of the mudsnail *Cipangopaludina cathayensis* (Gastropoda: Viviparidae). Mitochondrial DNA 16: 1–3. 10.3109/19401736.2014.971274 25319293

[B41] Yu DJ Xu L Nardi F Li JG Zhang RJ (2007) The complete nucleotide sequence of the mitochondrial genome of the oriental fruit fly, *Bactrocera dorsalis* (Diptera: Tephritidae). Gene 396: 66–74. doi: 10.1016/j.gene.2007.02.023 17433576 10.1016/j.gene.2007.02.023

[B42] Zhang DX Hewitt GM (1997) Insect mitochondrial control region: A review of its structure, evolution and usefulness in evolutionary studies. Biochemical Systematics and Ecology 25: 99–120. doi: 10.1016/S0305-1978(96)00042-7

